# miRNA-mRNA Correlation-Network Modules in Human Prostate Cancer and the Differences between Primary and Metastatic Tumor Subtypes

**DOI:** 10.1371/journal.pone.0040130

**Published:** 2012-06-29

**Authors:** Wensheng Zhang, Andrea Edwards, Wei Fan, Erik K. Flemington, Kun Zhang

**Affiliations:** 1 Department of Computer Science, Xavier University of Louisiana, New Orleans, Louisiana, United States of America; 2 IBM T. J. Watson Research, Hawthorne, New York, United States of America; 3 Department of Pathology, Tulane University Health Sciences Center and Tulane Cancer Center, New Orleans, Louisiana, United States of America; Università di Napoli Federico II, Italy

## Abstract

Recent studies have shown the contribution of miRNAs to cancer pathogenesis. Prostate cancer is the most commonly diagnosed cancer in men. Unlike other major types of cancer, no single gene has been identified as being mutated in the majority of prostate tumors. This implies that the expression profiling of genes, including the non-coding miRNAs, may substantially vary across individual cases of this cancer. The within-class variability makes it possible to reconstruct or infer disease-specific miRNA-mRNA correlation and regulatory modular networks using high-dimensional microarray data of prostate tumor samples. Furthermore, since miRNAs and tumor suppressor genes are usually tissue specific, miRNA-mRNA modules could potentially differ between primary prostate cancer (PPC) and metastatic prostate cancer (MPC). We herein performed an *in silico* analysis to explore the miRNA-mRNA correlation network modules in the two tumor subtypes. Our analysis identified 5 miRNA-mRNA module pairs (MPs) for PPC and MPC, respectively. Each MP includes one positive-connection (correlation) module and one negative-connection (correlation) module. The number of miRNAs or mRNAs (genes) in each module varies from 2 to 8 or from 6 to 622. The modules discovered for PPC are more informative than those for MPC in terms of the implicated biological insights. In particular, one negative-connection module in PPC fits well with the popularly recognized miRNA-mediated post-transcriptional regulation theory. That is, the 3′UTR sequences of the involved mRNAs (∼620) are enriched with the target site motifs of the 7 modular miRNAs, has-miR-106b, -191, -19b, -92a, -92b, -93, and -141. About 330 GO terms and KEGG pathways, including TGF-beta signaling pathway that maintains tissue homeostasis and plays a crucial role in the suppression of the proliferation of cancer cells, are over-represented (adj.p<0.05) in the modular gene list. These computationally identified modules provide remarkable biological evidence for the interference of miRNAs in the development of prostate cancers and warrant additional follow-up in independent laboratory studies.

## Introduction

MicroRNAs (miRNAs) are short (∼*22nt*), non-coding RNAs derived from genome-encoded stem loop precursors. As the crucial post-transcriptional regulators of gene expression in metazoans, miRNAs primarily bind to the 3′ UTR sequences of messenger RNAs (mRNAs), usually resulting in translational repression or mRNA degradation [1], [2]. It is estimated that ∼30% of human protein-coding genes are regulated by miRNAs, where each miRNA can target approximately 200 transcripts and more than one miRNA can converge onto a single mRNA target [2], [3]. Numerous studies have shown that aberrantly expressed miRNA*s* are likely to contribute to human diseases, including cancer [4], [5], [6], [7], [8], [9]. However, these small RNAs could not be the “cancer-drivers” [10] in the majority of cancer cases because the evidences for their mutations in sematic cells are still relatively rare [11], [12]. This indicates that miRNAs themselves may be regulated by other molecules such as transcription factors [13] and, in turn, cooperatively play roles in disease progression by amplifying or reducing the impact of the aberrations occurring in proto-oncogenes and tumor suppressor genes. It has been recognized that the interference of miRNAs with tumorigenesis is quite complicated and needs to be scrutinized by the network-based systems biology approaches.

To date, a number of algorithms have been developed to infer miRNA-mRNA modules or modular networks using the genome-wide transcription and sequence affinity information [14], [15], [16], [17]. Despite the diverse algorithmic designs and computational complexities, the flow schemes of these methods are fairly explicit and the definitions of a miRNA-mRNA module carry similar characteristics. A fundamental module generally consists of a set of co-expressed protein-coding genes and a miRNA which is significantly correlated with these genes in the expression level, or is a top predictor (among other regulators) for the mRNA-set-determined classification trees of the biological samples/“conditions” [18], [19], [20]. Such a one-to-many type of module can then be refined into a canonical miRNA-mRNA regulatory module where the expressions of miRNAs and mRNAs are in inverse relationship, and the complementary motifs of the miRNAs’ seed sequences exist in the 3′UTRs of the target genes (mRNAs). Two or multiple one-to-many modules can further be combined into a many-to-many module by identifying their intersections [16].

**Figure 1:**
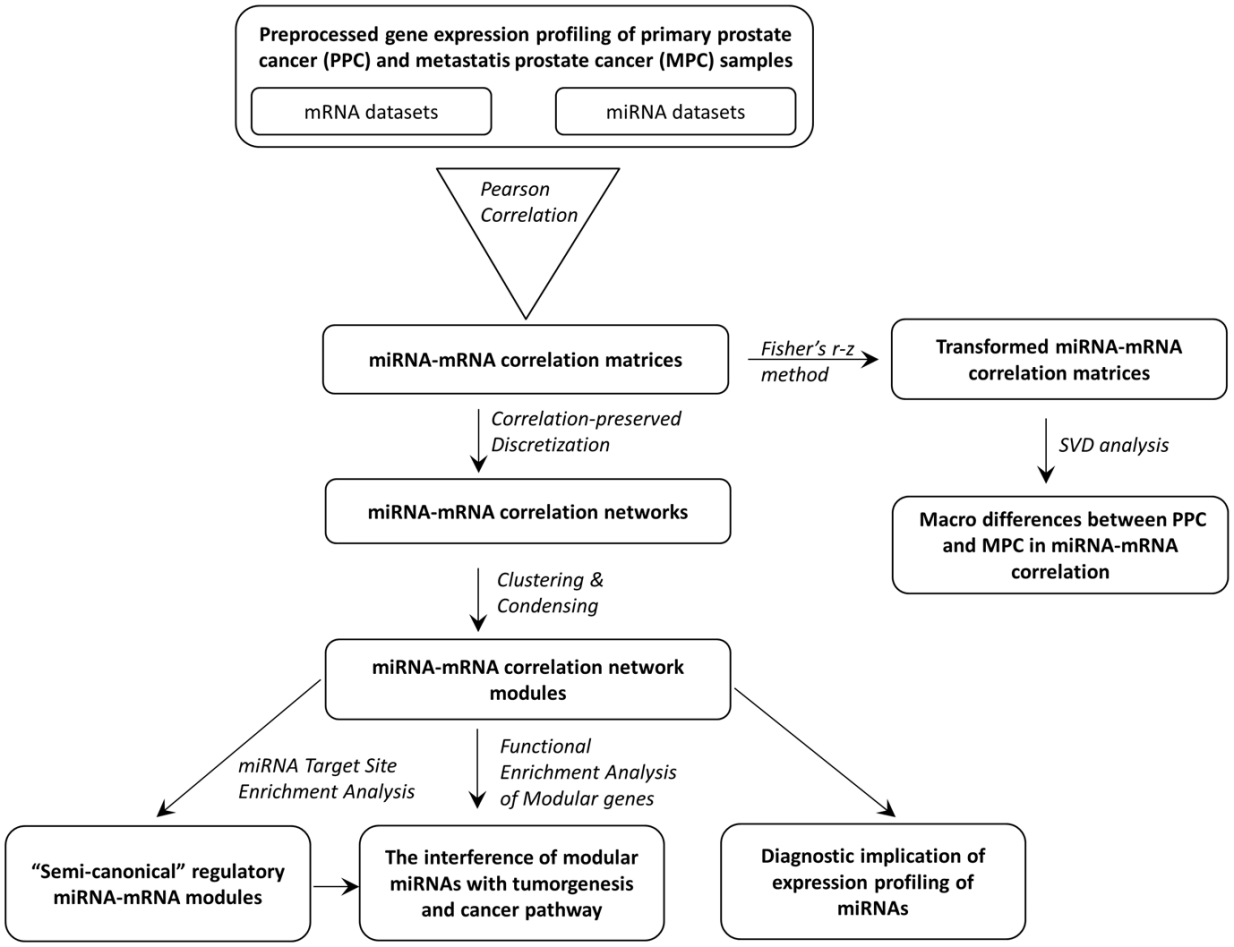
The schematic presentation of the study flow.

Prostate cancer is the most commonly diagnosed cancer and the second leading cause of cancer mortality in American men. Every year, more than 200,000 new cases are diagnosed and over 30,000 adult males die from this disease [21]. Fatal outcome often occurs when the local tumor infiltration has spread beyond the prostate gland and metastasized to lymph nodes and other organs. Unlike other major types of cancers, the genetic etiology of prostate cancer is rather complex and heterogeneous. No single gene mutation has been pinpointed in the majority of prostate tumors [12], [22], [23]. This implies that the expression profiling of genes, including non-coding miRNAs, may substantially vary across individual cases in different stages or subtypes of prostate cancer. The within-class variability, i.e. variability primarily due to the intrinsic differences in molecular genetic mechanism among sampled individuals of the same class, makes it possible to reconstruct or infer the disease specific correlation and regulatory (modular) networks using the high-dimensional microarray data of prostate tumor samples. In particular, because miRNAs and tumor suppressor genes are usually tissue specific [23], [24], miRNA-mRNA modules potentially differ between primary prostate cancer (PPC) and metastatic prostate cancer (MPC). Our study thus initiated from this perception and centered on the *in silico* identification and comparison of miRNA-mRNA modules in PPC and MPC. A recently released comprehensive database [12] provided us the required information for such a bioinformatics investigation. The obtained results provide remarkable biological insight into the interference of miRNAs in the development of prostate cancers. **Figure 1** summarizes the scheme of our study flow, and the details of each step are described in the Results and Method sections.

**Figure 2:**
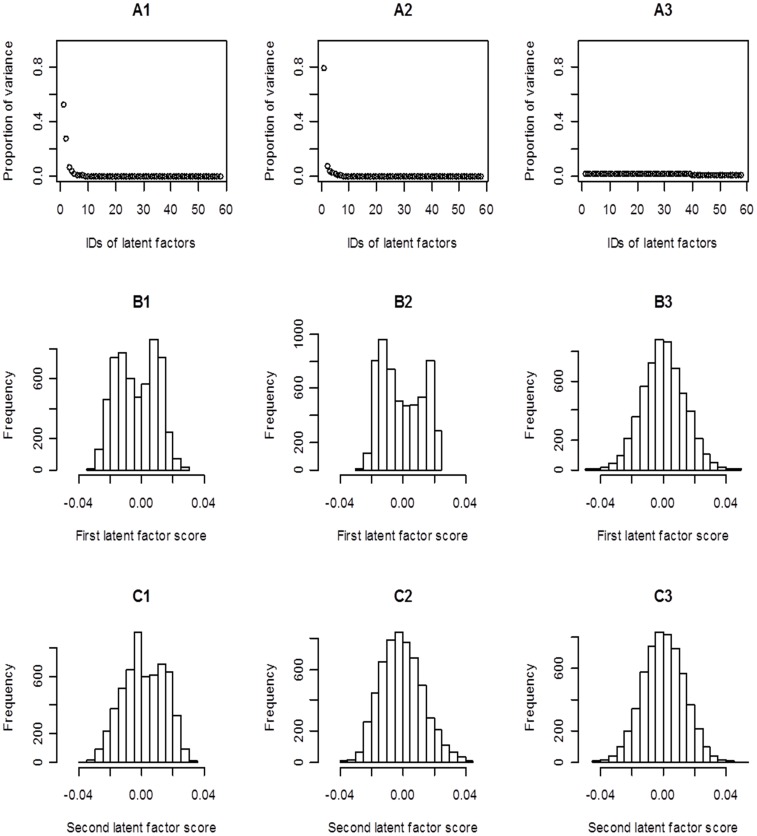
The singular value decomposition (SVD) analysis of miRNA-mRNA transcriptional correlation matrices. Before the decomposition, Pearson correlation coefficients were transformed with Fisher’s r-z method. **A1/B1/C1**: the results obtained from the primary prostate cancer (PPC) samples. **A2/B2/C2**: the results obtained from the metastatic prostate cancer (MPC) samples. **A3/B3/C3**: the results obtained from a random data structure generated by shuffling the PPC matrix.

**Table 1:** The summary of miRNA-mRNA correlation-network modules.

Module^a^	Gene number	Number of TF genes^b^	miRNA^c^
*p-modu-1-ps*	542	39	**-1**, -143, -145, **-221**, **-222**
*p-modu-1-ne*	113	7	-1, -143, -145, -221, -222
*p-modu-2-ps*	466	50	**-181c**, -224, -24, **-27a**
*p-modu-2-ne*	92	8	-181c, -224, -24, -27a
*p-modu-3-ps*	233	6	-7a, -7d, -7f, -7g, -7i, -126, -195, -98
*p-modu-3-ne*	6	0	-7a, -7d, -7f, -7g, -7i, -126, -195, -98
*p-modu-4-ps*	350	26	-146a, -150, **-223**
*p-modu-4-ne*	38	5	-146a, -150, -223
*p-modu-5-ps*	145	11	-106b, -141, -19a, -19b, -200c, -92a, -93
*p-modu-5-ne*	622	47	**-106b**, **-141**, **-19a**, **-19b**, **-200c**, **-92a**, **-93**
*m-modu-1-ps*	81	2	-17, -20a, -20b
*m-modu-1-ne*	15	0	-17, -20a, -20b
*m-modu-2-ps*	173	7	**-200a**, **-200b**
*m-modu-2-ne*	382	6	-200a, -200b
*m-modu-3-ps*	115	5	-15a, -26a, -29c
*m-modu-3-ne*	123	0	-15a, -26a, -29c
*m-modu-4-ps*	92	4	-7a, -7d, -7e, -7f, -98
*m-modu-4-ne*	138	2	-7a, -7d, -7e, -7f, -98
*m-modu-5-ps*	86	3	-107, -26b
*m-modu-5-ne*	182	3	-107, -26b

a The modules in primary prostate cancer (PPC) or metastatic prostate cancer (MPC) are marked with the prefix “*p-*” or “*m-*”. The positive or negative-connection modules are marked with the extension “*-ps*” or “*-ne*”. **^b^** Only the sequence-specific DNA binding transcription factors were counted. The TF genes have been annotated to one of the five GO terms: GO: 0003700 (sequence-specific DNA binding transcription factor activity), GO: 0003702 (RNA polymerase II transcription factor activity), GO: 0003709 (RNA polymerase III transcription factor activity), GO: 0016563 (transcription activator activity), and GO: 0016564 (transcription repressor activity). **^c^** The prefix “hsa-miR” are omitted in the IDs. For a miRNA, the statistical significance of the target site enrichment level in the list of the correlated modular genes was measured by the Fisher’s exact test in reference to the level of the entire gene set. The red bold font indicates p-value <0.01.

**Figure 3:**
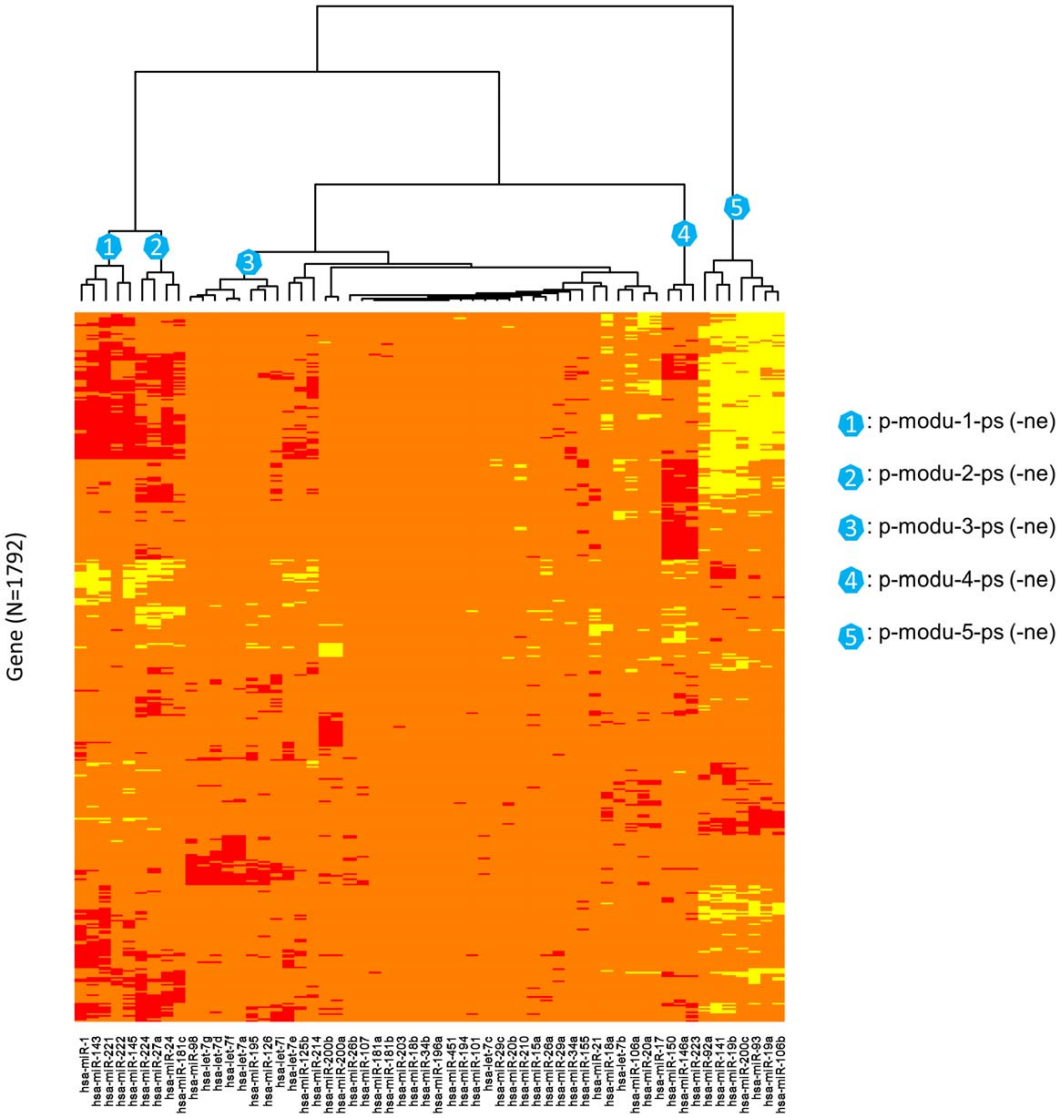
Identifying miRNA-mRNA correlation-network modules in primary prostate cancer (PPC) by the hierarchical clustering algorithm. Red: the top 1% positive correlations. Yellow: the top 1% negative correlations. Orange: pseudo or unconsidered correlations.

**Figure 4:**
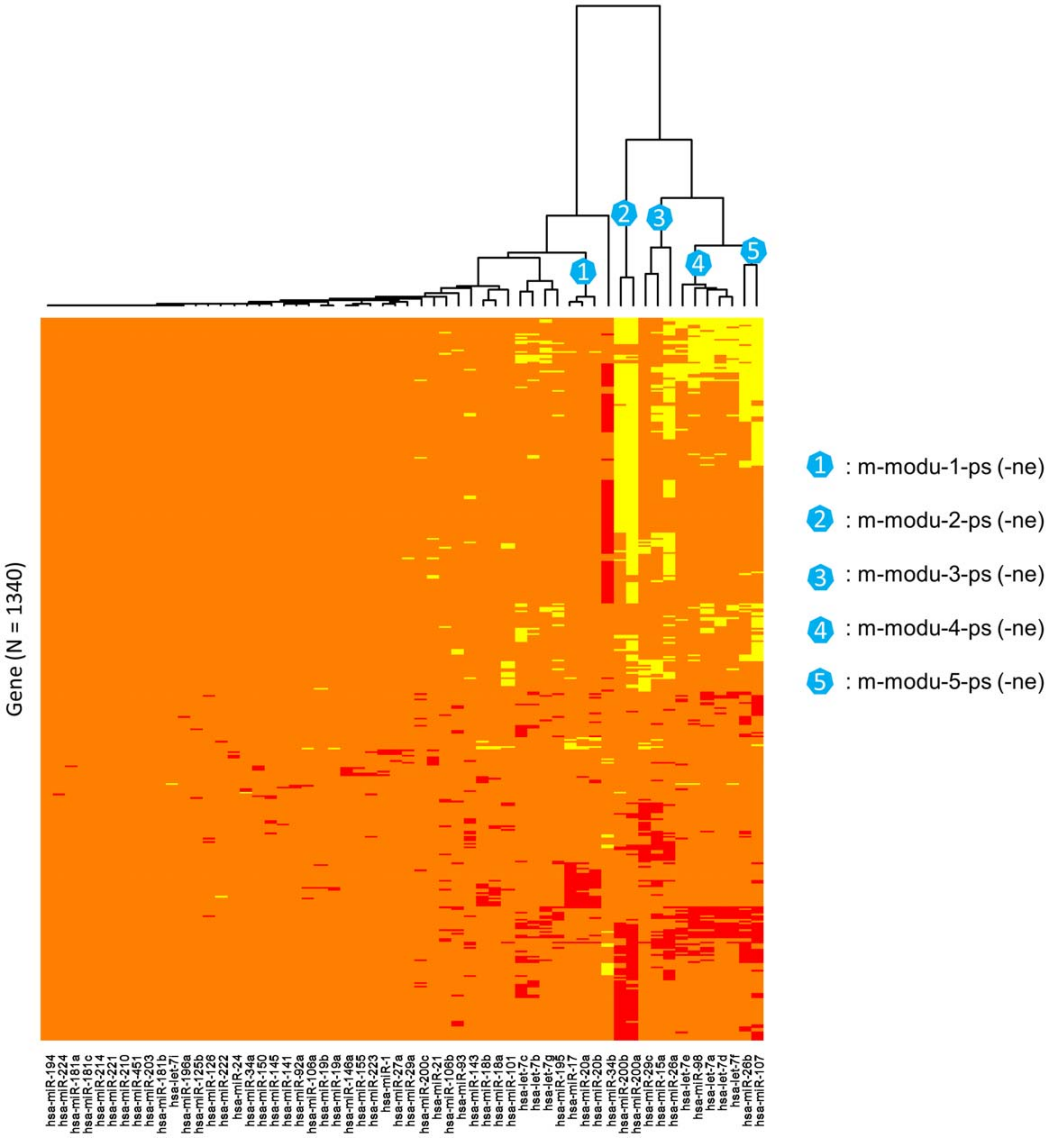
Identifying miRNA-mRNA correlation-network modules in metastatic prostate cancer (MPC) by the hierarchical clustering algorithm. Red: the top 1% positive correlations. Yellow: the top 1% negative correlations. Orange: pseudo or unconsidered correlations.

**Figure 5:**
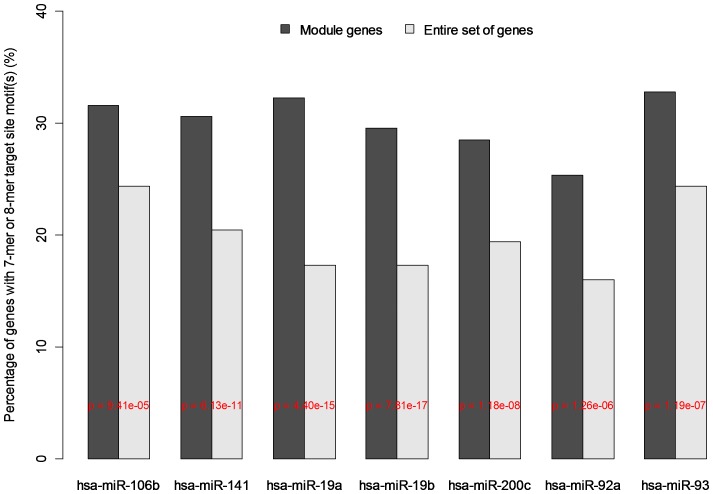
miRNA target site enrichment analysis for module *p-modu-5-ne.*

## Results and Discussion

### SVD Analysis of miRNA-mRNA Interaction Matrices

Before inferring the miRNA-mRNA network modules for PPC and MPC, we tried to obtain a general understanding of the differences between those two correlation matrices calculated from the miRNA/mRNA expression levels of the primary and metastatic tumor samples, respectively (see the Method section for details). However, due to the enormous number of potential miRNA-mRNA regulatory or co-expressed relationships and the complicated interplay among them, together with the noises introduced into the sampling and measurement process, the pairwise comparison of the corresponding matrix elements was too trivial to reach a conclusion. We circumvented this obstacle by employing a novel method. More specifically, we treated each correlation matrix as a pseudo dataset with the rows (mRNAs) as “observations” and the columns (miRNAs) as “features”, and characterized it by conducting SVD (Singular Value Decomposition) analysis [25], [26], [27], [28]. Considering the values of correlation coefficients were in the range of [-1, 1], we transformed the matrix entries using Fisher’s r-z method prior to the decomposition so that the results can be explained by the standard statistical theory.

**Figure 6:**
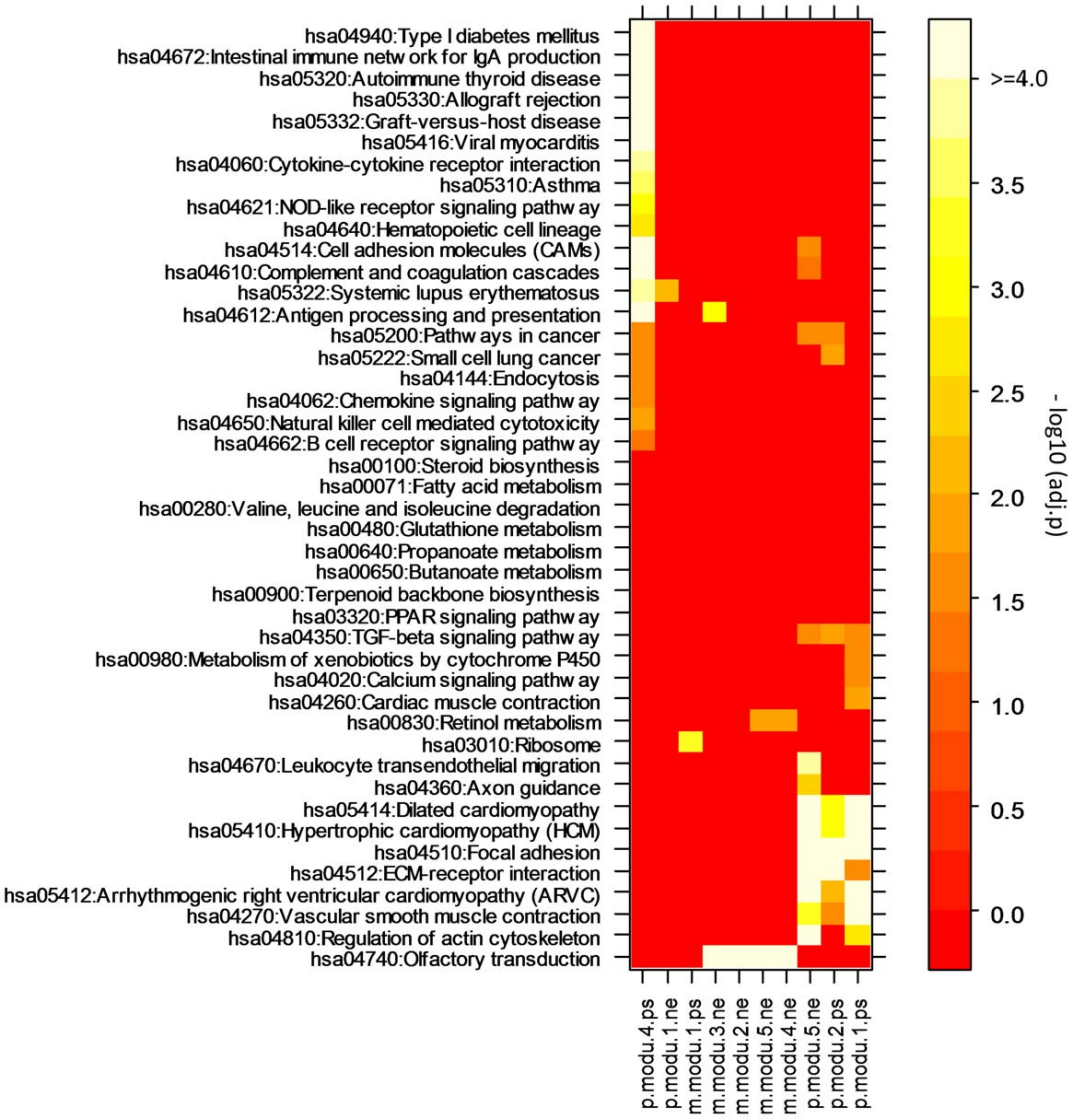
Over-represented KEGG pathways in the individual modular gene sets of 10 major miRNA-mRNA modules discovered for primary prostate cancer (PPC) or metastatic prostate cancer (MPC). The ordinary p-values were adjusted with BH method.

**Figure 7:**
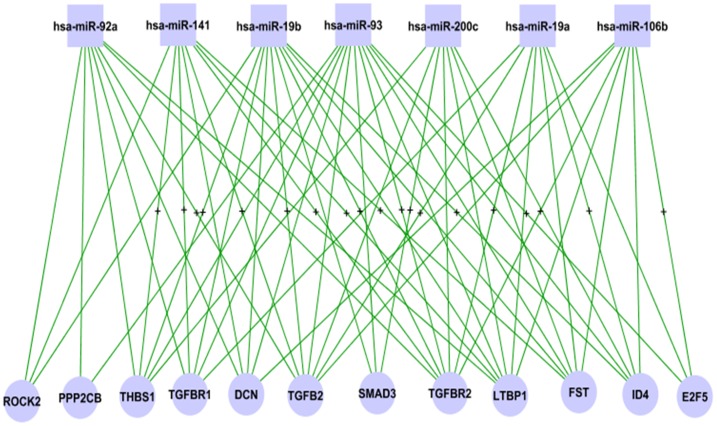
Modular miRNA-mRNA network for TGF-beta signaling pathway. The sub-network was extracted for modules *p-module-5-ne(ps)*. All the connections except for those related to E2F5 are negative. Signal + indicates that at least one miRNA-target-site motif exists in the 3′ UTR sequence of the connected mRNA (gene).

**Figure 8:**
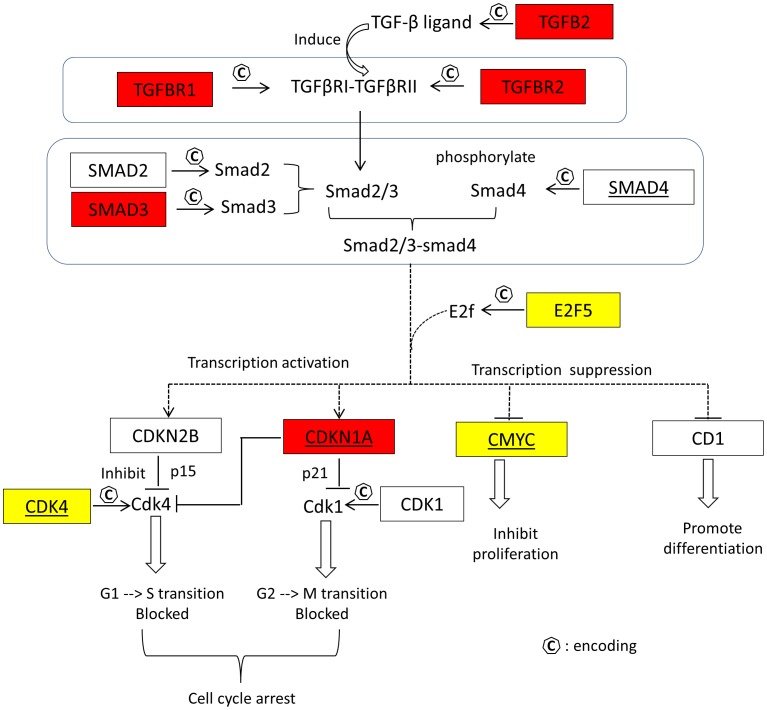
The interference of miRNAs in module *p-module-5-ne* in TGF-beta signaling pathway . The genes which have negative connections with the miRNAs in the modular network are shaded with red. Gene CDKN1A (albeit not in the module gene list) is also shaded with red because of the significant negative correlations (p<0.001) with hsa-miR-93, -106b and -200c at the expression level. Genes E2F5, MYC (CMYC) and CDK4, demonstrating an apparent pattern of positive transcriptional correlations with the modular miRNAs (**Figure 9**), are shaded with yellow.

**Figure 9:**
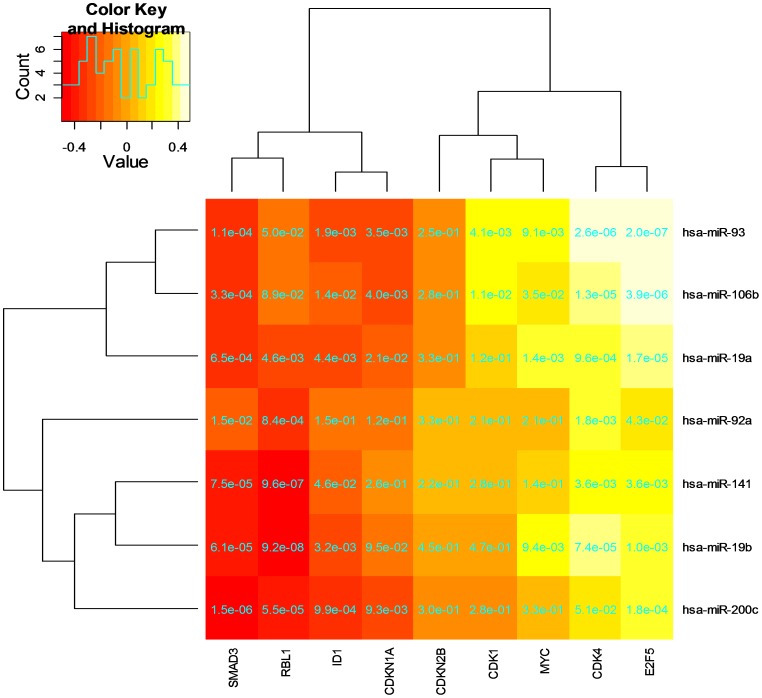
The transcriptional correlations of miRNAs in modules *p-module-5-ne (ps)* with TGF-beta signaling pathway downstream genes. The number presented on each cell is the p-value of the correlation for the corresponding miRNA and mRNA (gene).

This analysis led to two interesting findings. First, for both PPC and MPC correlation matrices, the leading latent factor can explain over half of the variance in data (**Figure 2** **-A1, -A2**), and the scores (in the first left singular vector) across genes (**Figure 2** **-B1, -B2**) show a clear two-peak distribution. Second, the PPC matrix differs from the MPC matrix by a substantial second latent factor that explains ∼20% of the variance and has an asymmetric score (in the second left singular vector) distribution (**Figure 2** **-C1**), which deviates from the symmetric counterpart for MPC (**Figure 2** **-C2**). The strength of this analysis is further highlighted by the fact that the observed patterns are not present in a random data set as shown in **Figure 2** **-A3**, **-B3 and C3**, where SVD analysis was conducted on a matrix generated by shuffling the rows and columns of the PPC matrix. While the biological implications need to be further investigated, these findings preliminarily confirmed our initial hypothesis that the dynamic interference of miRNAs in those two types of prostate cancer can be different and worth further exploration.

It is worth noting that, in preparing **Figure 2**, we used the information of 58 cancer miRNAs collected in [7]. However, the presented results largely held when the SVD analysis was conducted on the entire datasets (matrices). In addition, we recognize that the differences between the PPC and MPC can be determined by a core set of miRNAs. This perception is based on an additional analysis which showed that the patterns demonstrated in **Figure 2** also held on the sub-matrices with only the tumor suppressor miRNAs, but not on the sub-matrices containing the oncogene miRNAs exclusively. We will continue to investigate this problem in future research.

### miRNA-mRNA Modules in different Prostate Cancer Subtypes

Two miRNA-mRNA networks (PN and MN) were generated by a correlation-based method for PPC and MPC, respectively. In order to focus the analysis on the cancer-related miRNAs, the dimensions of PN and MN were further reduced as presented in the Method section. With the condensed networks as inputs, major cancer-related miRNA-mRNA modules were identified by a clustering analysis-based algorithm. Within an individual module, each miRNA or mRNA has at least two connections with the corresponding modular mRNAs or miRNAs.

As summarized in **Table 1** **,** **Figure 3** and **Figure 4**, we identified 5 miRNA-mRNA module pairs (MPs) for PPC and MPC, respectively. Each MP includes one positive-connection (correlation) module and one negative-connection (correlation) module. The number of miRNAs or mRNAs (genes) in each module varies from 2 to 8 or from 6 to 622. Most of the modules contain the sequence-specific DNA binding transcription factor (TF) genes [29] that may take roles as the mediators for the miRNA-mRNA connections. The two members (such as *p-modu-1-ps* and *p-modu-1-ne* in PPC) of each MP contain the same miRNAs but different mRNAs. Two modules of distinct MPs (such as *p-modu-1-ps* and *p-modu-2-ps* in PPC) consist of different miRNAs and varied (or partially overlapped) mRNA sets. Within a positive or negative-connection module (with “*-ps*” or “*-ne*” extension in the IDs), the correlations of the involved miRNAs and mRNAs at the expression levels are consistently positive or negative. Regardless of the connection type, the mRNA set in each module largely imitates a co-expressed gene cluster. Besides the four modules of the two MPs (*p-modu-3-ps* (*-ne*) and *m-modu-4-ps* (*-ne*)) in which most miRNAs belong to the let-7 family, the modules for MPC hardly overlap with the modules for PPC in terms of the involved miRNAs (**Table 1**) and protein-coding genes (**Table S1**). Advanced insights regarding the differences between PPC and MPC can be further inferred by a scrutiny of the biological implications of the identified modules.

Here we need to point out that the identified modules are not necessarily the canonical regulatory modules in which all the connections between miRNAs and genes are determined by the causal regulator-target relationships [30]. In fact, due to the unsolved problem in accurately recognizing the miRNA target sites [2], it is challenging to identify a nontrivial and exact canonical regulatory module. Nonetheless, we can expect to find a less strict regulatory module, called a “semi-canonical” regulatory module hereafter, where the relationship potentially determined by the miRNA-mediated mRNA degrading mechanism is predominant among the miRNA-mRNA connections. Based on this widely-accepted theory, semi-canonical regulatory modules, if exist, should be among the negative-connection modules, and then can be determined by the target site enrichment analysis. We did this exploration by establishing the miRNA-mRNA sequence affinity matrix and conducting Fisher’s exact test (see the Method section). As a result, we found that *p-modu-5-ne* **,** a module identified for PPC, was the only semi-canonical module where the 3′UTR sequences of the involved 622 mRNAs were significantly enriched (p<1.0E−4) with the target site motifs of the 7 modular miRNAs (hsa-miR-106b, -200c, -19b, -92a, -92b, -93, and -141) (**Figure 5**). This module, therefore, represents the most significant difference between PPC and MPC in terms of the observed miRNA-mRNA correlations. It suggests that the post-transcriptional regulation mediated by the documented cancer miRNAs directly contribute to the expression variability of the protein-coding genes across the tumor samples for PPC but not for MPC.

The implications of the identified modules need to be further inferred through the functional annotations of the modular genes, since there is a great biomedical interest in elucidating the relationships between the activity of modular miRNAs, as regulators or biomarkers, and the variability of the specific biological processes. Using the David database [31], we found that over 330 GO terms and KEGG pathways, including the TGF-beta signaling pathway, were over-represented (BH adj.p<0.05) with the 622 genes of the semi-canonical module *p-modu-5-ne*. The genes within other individual modules also demonstrated the significant functional similarity. For example, *m-modu-4-ne* **,** a negative-connection module discovered for MPC, was enriched with the genes in the GO:0007186∼G-protein coupled protein signaling pathway (BH adj.p  = 1.14E−40). **Table S2** summarized the functional enrichment analysis results of the modular genes**.**

We also examined the differences between these two prostate cancer subtypes by studying the distributional profile of the KEGG pathways over-represented in the individual modular gene set. As shown in **Figure 6**, module *p-modu-4-ps* for PPC is unique among the identified modules regarding the functions of the modular genes. About two dozen of the pathways, such as type I diabetes mellitus, are over-represented in its gene list and most of them are related to disease process and/or immune response. Three miRNAs (hsa-miR-146a, -150 and -223) are included in this module. The involvement of miR-146a in the immune response has been widely investigated. [32] showed that miR-146a is a modulator of IL-2 and activation-induced cell death in lymphocytes. [33] reported that it mediates an inflammatory circuit in Alzheimer disease and stressed human brain cells. Another study [34] also demonstrated that HSV-1 infection in human brain cell can induce the expression of miR-146a. Apparently, these findings not only indicate that miR-146a takes a functional role in immune response as a regulatory factor, but also suggest that its dynamic activity (expression), in some specific contexts, may just serve as the passenger of disease and/or immune processes [10]. This is exactly the main message conveyed by the module *p-modu-4-ps* where the positive correlations between the modular miRNAs and genes could not be simply explained by a regulator-target mechanism.

Olfactory receptors (ORs) are expressed not only in the sensory neurons of the olfactory epithelium, but also in various other tissues where their potential functions are largely unknown. In a recent publication, the authors reported that the activation of an olfactory receptor (PSGR) inhibits proliferation of prostate cancer cells [35]. The results from our analysis show that olfactory transduction (pathway) is over-represented in the gene list of 4 major modules (*m-modu-2-ne*, *m-modu-3-ne*, *m-modu-4-ne*, *m-modu-5-ne*) in MPC (**Figure 6**). The involved miRNAs include hsa-miR-200a/-200b, hsa-miR-15a/26a/29c, hsa-miR-7a/-7e/-7f/-98, and hsa-miR-107/-26b. Although the miRNA set in *p-modu-3-ps (-ne)* largely overlapped with that in *m-modu-4-ne*, no module in PPC has a functional relationship with olfactory conduction. Therefore, we speculate that the activation of PSGR and the (direct or indirect) association with miRNAs are only confined to MPC.

Focal-adhesion kinase (FAK) is an important mediator for growth-factor signaling, cell proliferation, cell survival and cell migration. Mouse models have shown that FAK expression is increased in human tumors [36]. A recent study demonstrated that focal adhesion controls prostate cancer progression [37]. In this study, we found that miRNAs interfere in the transduction of FAK signaling, thus may take roles in cancer development. As shown in **Figure 6**, focal adhesion (together with 6 related KEGG pathways such as ECM-receptor interaction) is over-represented in the gene lists of 3 major modules identified for PPC (*p-modu-1-ps*, *p-modu-2-ps*, *p-modu-5-ne*). Opposite to the cases of olfactory transduction for MPC, the association of by a hierarchical clustering algorithm FAK signaling with miRNAs seems limited to PPC. These findings indicate another major difference between the two cancer subtypes. Experimental investigation on this issue could be promising for the diagnosis of prostate cancer.

### Potential Interference of miRNAs in TGF-beta Signaling Pathway

The transforming growth factor-beta (*TGF*-*beta*) maintains tissue homeostasis and plays a crucial role in the suppression of the proliferation of cancer cells [23], [38]. As mentioned above, the TGF-beta signaling pathway is over-represented in the gene (mRNAs) set of the semi-canonical regulatory module, *p-modu-5-ne*. Of the 622 modular genes, a dozen of them encode proteins in the pathway. These 12 genes demonstrate 56 negative-connections with the 7 modular miRNAs, and nearly one third of the connections are compatible with the potential regulator-target relationships determined by the sequence affinity information (**Figure 7**). Based on this finding and the theories described in [23] (pages 198–224), we generated a hypothesized model (**Figure 8**) to show the interference of the modular miRNAs with the TGF-beta signaling and the proliferation of cancer cells. In **Figure 8**, the genes negatively correlated with the miRNAs in the modular network are highlighted in red. Gene CDKN1A is also marked in red because of its significant negative correlations (p<0.001) with hsa-miR-93, -106b and −200c at the expression level. Genes E2F5, CMYC (MYC) and CDK4 show an apparent pattern of positive transcriptional correlations with the modular miRNAs and are highlighted in yellow. The relationships presented in **Figure 8** suggest that the modular miRNAs interfere with the disease process of the primary prostate cancer by an oncogenic mechanism in the measured PPC samples. More specifically, the expression of those miRNAs inversely regulates the transcript intensity of two GF-beta genes, TGFBR1 and TGFBR2, and a cancer suppression gene SMAD3. Then, through the activation or inactivation of the Smad2-3/Smad4/E2f complex, this effect is reflected on the expression levels of CDKN1A and CMYC, and finally influences the cell cycle arrest and cell proliferation inhibition.

### Expression Variability of miRNAs in Module *p-modu-5-ne*

As discussed above, in *p-modu-5-ne* **,** the identified miRNAs directly regulate the transcript intensities of the modular mRNAs in the PPC samples. In this regard, it is important to elucidate the etiology of the biological variability (across tumor samples) of the miRNA expression levels that determine the miRNA-mRNA connections. First, we noted that most of the identified miRNAs, including hsa-miR-19b, -92a, -93 and -106b, have been reported as the targets of the transcription factors of the E2F family [13], [39]. Meanwhile, we also observed the positive connections between these miRNAs and E2F5 at the expression levels (**Figures 8** **and** **9**). Therefore, there was a possibility that the biological variability of the miRNAs was due to the regulation by E2F5. However, such a mechanism needs to be further investigated since the actual picture of the regulation and/or mutation of the TF gene itself is still not clear. Next, we asked if the modular miRNA expression levels were related to the progression of prostate cancer. To investigate this issue, we grouped the 98 PPC samples on the expression levels of the 7 modular miRNAs by a hierarchical clustering algorithm and compared the result with the Gleason score-based classes. No association was found between the two partitions. Finally, we proposed and tested a hypothesis that the biological variability of the miRNAs was sourced from the regulation by the protein-coding genes (including cancer genes) on which mutations sporadically occurred in individual tumor samples. By clustering analysis, we firstly generated two partitions of the 98 PPC samples, respectively based on the expression levels of all the 7 module miRNAs and the transcript intensities of 34 potential cancer-driving genes in the tumor samples as shown in the figure-1 of [12]. Then by a Chi-square test, we found the association between the two partitions was extremely significant (p<0.001), indicating our hypothesis can be confirmed in this way.

## Materials and Methods

### Data Sets

The microarray data was published in the Gene Expression Omnibus (GEO, a MIAME compliant database) repository with accession number GSE21032 [12], [40]. Among the 218 biological samples included in the super-series, 98 primary tumors, 13 metastatic tumors and 28 normal prostate tissue samples (N  = 139) have both miRNA and mRNA expression profiles. Rigorous criteria have been applied in selecting tumors for the genomic analysis [12]. The gene (mRNA) expression profiles were measured with Affymetrix Human Exon 1.0 ST Array [probe set (exon) version], and the images were quantified using the GeneChip Operating Software (GCOS) version 1.4. The Raw data were processed by Aroma Affymetrix. The standard RMA background adjustment and quantile normalization were performed. The miRNA expressions were measured on the platform of Agilent-019118 Human miRNA Microarray 2.0 G4470B, and the images were quantified using Agilent Feature Extraction version 9.5. The data were normalized with between-array variance stabilization normalization (VSN) after excluding the microRNAs not present in over 80% of the profiled samples. In this study, the mRNA data (the downloaded Series Matrix File) was further simplified by following procedure. First, we adapted the gene expression values that originally centered on Affymatrix_Exon_Gene_IDs (Affy_IDs) to the official gene symbols by calculating the mean for a gene with two or multiple Affy_IDs. Second, the adapted gene expression values were transformed into log2 scale. Finally, we filtered out the genes that lack of expression variability across the 139 arrays. With a cutoff of a two-fold change from the maximum expression intensity to the minimum one, ∼ 5730 genes passed the filter. Therefore, the final miRNA datasets contains 5730 genes and 377 miRNAs. While the information of the 28 normal tissue samples was considered in the data preprocessing to dilute the potential noise in the microarray experiments, we focused on the PPC and MPC samples in the following SVD analysis and miRNA-mRNA module identification.

### Computation of miRNA-mRNA Correlation Matrices

We used a computationally intensive method to estimate the expression correlation between a miRNA and an mRNA. The calculation of Pearson correlation was repeated 1000 times. In each run, 80% of the randomly-selected PPC or MPC samples were involved. The final estimation was obtained by averaging the results over the 1000 replications.

### Generation of miRNA-mRNA Correlation Networks

We respectively generated two miRNA-mRNA correlation networks, PN and MN, for PPC and MPC by discretizing the expression correlation matrices with 5730 genes (mRNAs) as rows and 377 miRNAs as columns. More specifically, we filled in both matrices with 1 for the top 1% of the positive miRNA-mRNA correlations, -1 for the top 1% of the negative correlations, and 0 for the rest of the entries. Thus, a non-zero element represents a positive or negative correlation for a pair of miRNA and mRNA. A correlation defined in such a way corresponds to a miRNA-mRNA connection with the BH adjusted p-value <0.008 (or <0.065) for PPC (or MPC).

### Identification of miRNA-mRNA Modules

Both discretized correlation matrices were condensed by excluding the miRNAs lack of recorded relationships with cancers and the genes of no connection with the cancer-related miRNAs. That is, the columns corresponding to the miRNAs not included in the documented list in [7] were firstly removed, and then the rows (genes) which did not contain at least two non-zero entries were excluded. The condensed PN and MN thus contain 58 columns, and 1792 and 1340 rows, respectively. With the refined network matrices as inputs, two heatmaps were generated respectively for PPC and MPC by applying the function “*heatmap.2*” in the R package “gplots”. The layout of miRNAs and mRNAs in the heatmaps were based on a two-way hierarchical clustering analysis with *Manhattan* distance and *Ward* method as the arguments. Taking PPC as an example (**Figure 3**), we identified the miRNA-mRNA modules via the following three steps. (1) Based on the dendrogram and the miRNA-mRNA connection patterns shown on the heatmap, five modular miRNA subsets (clusters) were visually determined. (2) For each of the miRNA subsets, the positive or negative connections with mRNAs were collected into a couple of 2-column topology matrices, respectively. (3) A miRNA-mRNA module pair was identified from the outputs of step (2) after dropping the mRNAs with only one (positive or negative) connection. Figures of the modular networks were produced by Cytoscape 2.8.1 [41].

### Target Site Enrichment Test

Using a lab-owned R program with the core being the *matchPattern*() function in the Bioconductor *Biostrings* [42], [43], we identified the 7-mer and 8-mer miRNA target site motifs on the 3′ UTR sequences (retrieved from hg-18) of the genes measured in the employed microarray data. The binary miRNA-mRNA sequence affinity matrix (A) was then generated in a way such that an element (A_ij_) of value 1 indicated the existence of target site motif (s) for the j^th^ miRNA in the 3′ UTR sequence of the i^th^ mRNA. For a miRNA, the statistical significance of the target site enrichment level in the list of the correlated modular genes was measured by the Fisher’s exact test in reference to the level of the entire gene set.

## Supporting Information

Table S1 **The summary of miRNA-mRNA correlation-network modules.**
(TXT)

Table S2 **The summary of functional enrichment analysis of gene lists in the identified miRNA-mRNA correlation-network modules.** The BH adjusted p-values are listed. NA indicates that adj.p is >0.05 or no gene in the module has been annotated to the GO terms (or the KEGG pathway).
(TXT)
